# Analgesic effect of subcutaneous injection of different concentrations of methylene blue after hemorrhoidectomy: A retrospective study

**DOI:** 10.3389/fsurg.2023.1132277

**Published:** 2023-04-03

**Authors:** Qing Long, Jun Li, Yan Li

**Affiliations:** ^1^ Department of Traditional Chinese Medicine, The Affiliated Hospital of Southwest Medical University, Luzhou, China; ^2^ Department of Dermatology, Traditional Chinese Medicine Hospital Affiliated to Southwest Medical University, Luzhou, China

**Keywords:** subcutaneous injection, methylene blue, different concentrations, postoperative pain, hemorrhoidectomy

## Abstract

**Objective:**

Subcutaneous injection of methylene blue around the anus may help reduce postoperative pain. However, the concentration of methylene blue is still controversial. Therefore, Our study aims to investigate the efficacy and safety of different methylene blue injected concentrations subcutaneously in pain treatment after hemorrhoidectomy.

**Methods:**

A total of 180 consecutive patients with grade III or IV hemorrhoids from March 2020 to December 2021 were reviewed. All patients underwent hemorrhoidectomy under spinal anesthesia and were divided into three groups. Group A received subcutaneous injection of 0.1% methylene blue after hemorrhoidectomy, group B received subcutaneous injection of 0.2% methylene blue, and Group C did not received subcutaneous injection of methylene blue. The primary outcome measures were the visual analog scale (VAS) pain score on postoperative days 1, 2, 3, 7, 14, and total analgesic consumption within 14 days. Secondary outcomes were complications after hemorrhoidectomy, including acute urinary retention, secondary bleeding, perianal incision edema, and perianal skin infection, and the Wexner scores used to assess the level of anal incontinence at one and three months after surgery.

**Results:**

There was no significant difference among three groups in sex, age, course of the disease, hemorrhoid grade and the number of incisions, and there was no significant difference in the volume of methylene blue injected between group A and group B. The VAS pain score and total analgesics consumption within 14 days in group A and group B were significantly lower than those in group C, but the differences between group A and group B were not statistically significant. The Wexner scores of group B were significantly higher than those of group A and group C one month after the operation, but the differences between group A and group C were not statistically significant. In addition, the Wexner score among three groups decreased to zero at three months after operation. There was no significant difference in the incidence of other complications among three groups.

**Conclusion:**

The perianal injection of 0.1% methylene blue and 0.2% methylene blue have a similar analgesic effect in pain treatment after hemorrhoidectomy, but 0.1% methylene blue has higher safety.

## Introduction

Hemorrhoids are one of the most common anorectal diseases. According to the results of an epidemiological survey on common anorectal diseases of urban residents conducted in China from 2013 to 2014, the adults who reported having anorectal diseases accounted for 51.14% of the total survey population, and the incidence rate of hemorrhoids among anorectal diseases was the highest (50.28%) (1) Hemorrhoidectomy is generally advocated for patients with grade III or IV hemorrhoids (2). However, postoperative incision pain is very common and becomes an important reason for patients to refuse surgery (3). Postoperative pain reduces patients' acceptance and satisfaction with surgery, affecting wound healing and increasing hospitalization time and expenses.

In clinical practice, oral or intravenous opioids, nonsteroidal anti-inflammatory drugs, and other multimodal analgesia are often used to treat pain after hemorrhoidectomy (4), but many patients still feel obvious pain after surgery (5). Methylene blue can prevent nerve conduction and has strong analgesic, anti-inflammatory, and neurophilic properties (6). In recent years, methylene blue has been used to treat post-hemorrhoidectomy pain (7), postherpetic neuralgia (8), intractable anal pruritus (9), and other diseases. However, the concentration of methylene blue is still not uniform (6–9). There have been few reports on the effects of different concentrations of methylene blue on hemorrhoidectomy pain. Therefore, this retrospective study aimed to evaluate the efficacy and safety of subcutaneous injection of methylene blue at different concentrations for pain treatment after hemorrhoidectomy.

## Materials and methods

### Participants

This was a single-center retrospective study. We followed the retrospective observational study design. The ethics committee approved this study at the Affiliated Hospital of Southwest Medical University. We reviewed consecutive patients who underwent hemorrhoidectomy under spinal anesthesia. The same surgical team performed surgery from March 2020 to December 2021, and data from the electronic medical record system and prescription records were collected. The inclusion criteria were 18–65 years old, diagnosed with mixed hemorrhoids, grade III/IV hemorrhoids (Goligher's classification), and underwent hemorrhoidectomy under spinal anesthesia. The exclusion criteria included the following: concurrent additional anorectal diseases (e.g., perianal abscess, anal fistula, anal incontinence); a history of cardiac insufficiency; hepatic insufficiency; renal insufficiency; diabetes mellitus; coagulation disorders; peptic ulcer disease; incomplete perioperative clinical data.

### Methods

**Figure 1:**
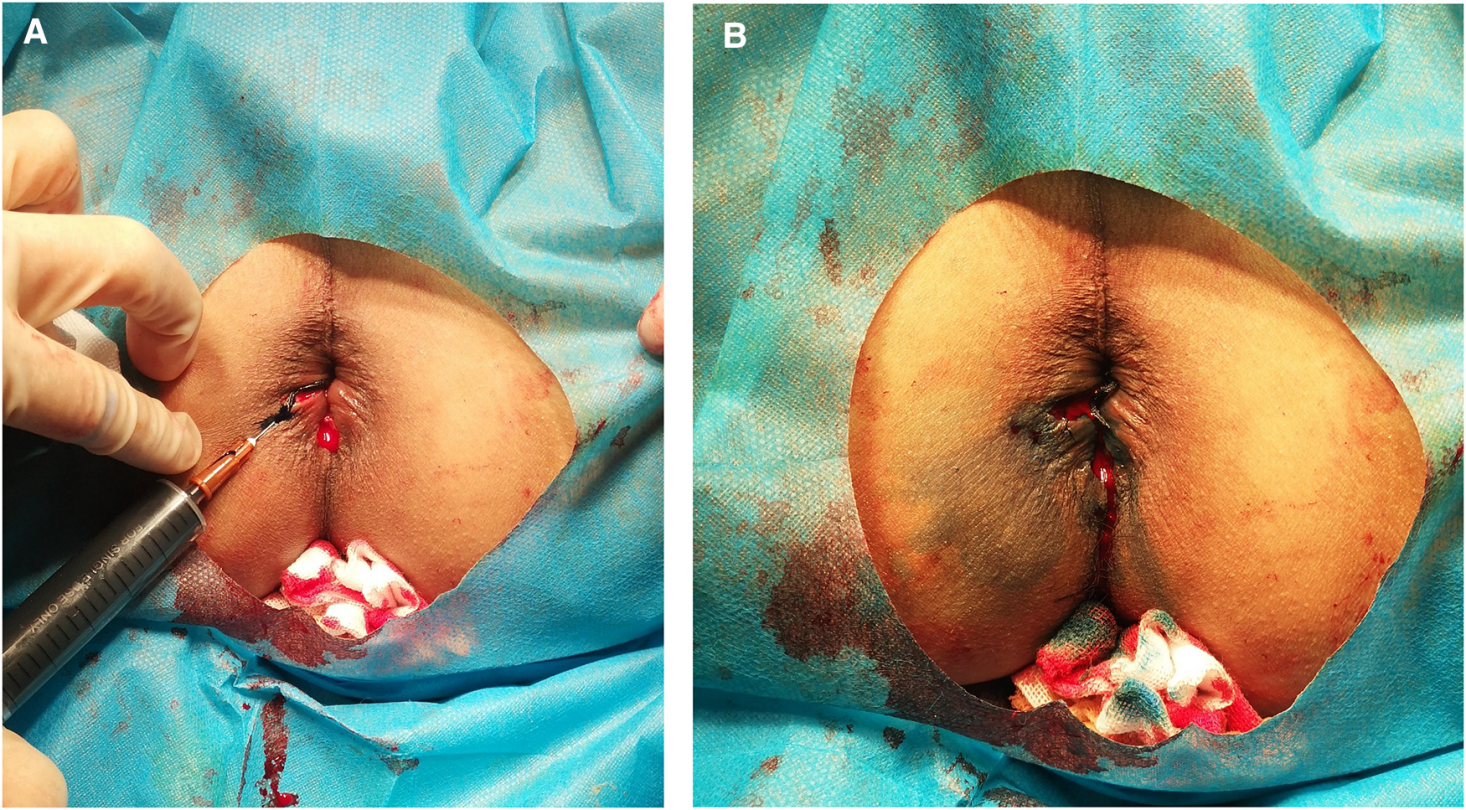
The technique of methylene blue injection. (**A**) Injecting from the distal end of the incision to the dentate line. (**B**) Incision after injection.

We reviewed a total of 180 patients who underwent hemorrhoidectomy under spinal anesthesia. The operations were performed by colorectal surgeons with senior professional titles according to standard techniques described by Milligan and Morgan (10). After the hemorrhoidectomy, 0.1% or 0.2% methylene blue was injected subcutaneously with a skin test needle at the edge of the perianal incision in Group A(*n* = 60) and Group B (*n* = 60), while Group C (*n* = 60) did not received subcutaneous injection of methylene blue. Group A received 0.1% methylene blue subcutaneously (1% methylene blue 1 ml + 0.1% ropivacaine 4 ml + 0.9% saline 5 ml) and group B received 0.2% methylene blue subcutaneously (1% methylene blue 2 ml + 0.1% ropivacaine 4 ml + 0.9% saline 4 ml). The total volume of injection was not more than 10 ml. Methylene blue was injected from the distal end of the incision to the level of the dentate line (Figure 1A). Methylene blue was injected with a 26-gauge needle from the distal end of the wound into the level of the dentate line. The injection site includes the cutaneous margins of the wound and the bed of the wound. The injection depth should not be too deep or too shallow to prevent the drug from entering the muscle or penetrating the skin. The standard was that the skin in the injection area was blue (Figure 1B). After injection of methylene blue, massage the injection site thoroughly so that the medication is evenly distributed under the skin. Postoperative management included stool control for 24 h, intravenous drip of antibiotics (cefuroxime) to prevent infection, clean anus with warm water sitz bath, and change of dressing after defecation. When the patient has constipation symptoms, oral laxatives (lactulose oral liquid) would be used to reduce incision pain during defecation. When the pain of the patient was intolerable, the oral analgesic nimesulide dispersible tablets (0.1 g/tablet) were given and the dose was recorded.

### Data collection

We extracted sex, age, course of the disease, hemorrhoid grade, the number of incisions, and the volume of methylene blue injected, the visual analog scale (VAS) pain score, complications and the Wexner incontinence scores (Wexner scores). Operationrelated variables from the electronic medical records' system and analgesics consumption information from the prescription monitoring program were collected. The primary outcome measures were the visual analog scale (VAS) pain score on postoperative days 1, 2, 3, 7, 14, and total analgesic consumption within 14 days. The VAS evaluates the intensity of pain from 0 (no pain) to 10 (very severe pain). Secondary outcomes were complications after hemorrhoidectomy, including acute urinary retention, secondary bleeding, perianal incision edema, perianal skin infection, and the Wexner scores used to assess the level of anal incontinence at 1 and 3 months after surgery. Two independent researchers analyzed all data.

### Statistical analysis

SPSS22.0 statistical software was used to process the data. The enumeration data are presented as counts (%), and the chi-square test or Fisher's exact test was used to compare differences and calculate *p*-values. The measurement data were expressed as mean ± standard deviation ($\bar{x}\pm s$). The data of three groups were compared by one-way ANOVA. When there was a significant difference among the four groups, the Bonferroni test was used for pairwise comparisons. The difference was considered significant when *P* < 0.05.

## Results

There was no significant difference among three groups in sex, age, course of the disease, hemorrhoid grade and the number of incisions, and there was no significant difference in the volume of methylene blue injected between group A and group B, as shown in Table 1.

**Table 1:** Comparison of patient demographics and clinical aspects among three groups.

Group	Group A	Group B	Group C	*P*-value
Mean age (years)	42.85 ± 11.75	41.81 ± 10.89	38.51 ± 11.11	0.096
Female/male	25/35	27/33	24/36	0.853
Courses of disease (years)	3.90 ± 3.27	4.15 ± 2.46	4.91 ± 2.90	0.138
Hemorrhoids grades (III/IV)	51/9	53/7	50/10	0.730
The number of incisions	2.96 ± 0.90	3.00 ± 0.73	2.98 ± 0.92	0.976
The volume of methylene blue injected(ml)	4.60 ± 1.01	5.43 ± 7.93	/	0.145

Mean age, courses of disease, the number of incisions, and the volume of methylene blue injected are presented as the mean ± standard deviation.

The VAS pain score on postoperative days 1, 2, 3, 7, and 14 and the total consumption of analgesics within 14 days in group A and group B were significantly lower than those in group C, but the differences between group A and group B were not statistically significant, as shown in Table 2. There was no significant difference in complications among three groups, including urinary retention, secondary bleeding, perianal incision edema, and skin infection. The Wexner scores of group B were significantly higher than those of group A and group C one month after the operation, but the differences between group A and group C were not statistically significant. In addition, the Wexner score among three groups decreased to zero at three months after operation, as shown in Table 3.

**Table 2:** Comparison of postoperative visual analog scale (VAS) for pain and total analgesic consumption over 14 days among three groups.

Group	Group A	Group B	Group C	*P*	*P*	*P*	*P*
				value	AvsB	AvsC	BvsC
VAS (1d)	4.01 ± 0.98	3.86 ± 0.98	4.48 ± 0.65	0.001	1.000	0.013	0.001
VAS (2d)	3.10 ± 0.81	3.06 ± 0.82	3.50 ± 0.96	0.011	1.000	0.038	0.021
VAS (3d)	2.63 ± 0.75	2.51 ± 0.87	3.05 ± 0.83	0.001	1.000	0.018	0.001
VAS (7d)	2.10 ± 1.00	1.90 ± 0.75	2.51 ± 0.92	0.001	0.677	0.037	0.001
VAS (14d)	0.96 ± 0.75	0.95 ± 0.87	1.40 ± 0.80	0.003	1.000	0.012	0.008
Total analgesic consumption within 14 days (g)	0.69 ± 0.19	0.65 ± 0.20	0.78 ± 0.17	0.001	0.695	0.031	0.001

Postoperative visual analog scale (VAS) for pain and total analgesic consumption over 7 days are presented as the mean ± standard deviation.

**Table 3:** Comparison of acute urinary retention, secondary hemorrhage, perianal incision edema, perianal skin infection, the Wexner score at one and three months after the operation among three groups.

Group	Group A	Group B	Group C	*P*	*P*	*P*	*P*
				value	A vs B	A vs C	B vs C
Acute urinary retention	6 (10.00%)	5 (8.33%)	6 (10.00%)	0.937	/	/	/
Secondary hemorrhage	0 (0%)	0 (0%)	0 (0%)	/	/	/	/
Perianal incision edema	12 (20.00%)	11 (18.33%)	9 (15.00%)	0.766	/	/	/
Perianal skin infection	0 (0%)	0 (0%)	0 (0%)	/	/	/	/
The Wexner score at one month after the operation	0.35 ± 0.57	0.70 ± 0.80	0.10 ± 0.30	<0.001	0.005	0.071	<0.001
The Wexner score at three months after the operation	0	0	0	/	/	/	/

Acute urinary retention, secondary hemorrhage, perianal incision edema,and perianal skin infection are presented as N (percentage). The Wexner score at one month after the operation is presented as the mean ± standard deviation.

## Discussion

Although stapled hemorrhoidopexy (11) and Doppler-guided hemorrhoid artery ligation (HAL) (12) can be used to treat hemorrhoids, several systematic reviews have compared the treatment effects of stapled hemorrhoidopexy, HAL, and hemorrhoidectomy. The results show that compared with hemorrhoidectomy, stapled hemorrhoidopexy has more short-term benefits, such as less pain, faster recovery, shorter hospital stay, shorter time of returning to normal activities, and higher patient's satisfaction, but the incidence of postoperative prolapse and the re-intervention rate of prolapse are higher in patients undergoing stapled hemorrhoidopexy (13, 14). Although HAL has less bleeding after operation, the number of patients requiring r-emergency surgical intervention is significantly reduced, and the recovery is faster, but the recurrence rate is high (15). Therefore, although hemorrhoidectomy has some disadvantages, such as long postoperative pain, pain period, and recovery period, the treatment effect of this method is clear, and the long-term success rate is high. It is still the preferred surgical treatment and “gold standard operation” for patients with grade III-IV hemorrhoids (16, 17). The pain after hemorrhoidectomy is related to many factors, such as spasm of the anal sphincter and puborectal muscle, delayed wound healing, acute local inflammatory reaction caused by tissue trauma, surgical technique, stool type, and subjective perception of patients (18–20). The unsatisfactory analgesia effect after a hemorrhoid operation limits the activity ability and self-care ability of patients, reduces their quality of life (21), prolongs the hospitalization time, increases the demand for opioid analgesia (22), and may increase myocardial ischemia, arrhythmia, thromboembolism, urinary retention and intestinal obstruction (23). Therefore, it is essential to minimize the pain after hemorrhoidectomy.

In the clinic, multimodal analgesia methods are often used to treat incision pain after hemorrhoid surgery, including opioid analgesics, nonsteroidal anti-inflammatory drugs (NSAIDs), metronidazole, flavonoids, laxatives, local anesthetics, botulinum toxin, and local calcium channel blockers (24, 25). However, despite the standard pain management, some patients still have problems in postoperative pain control (22).

Methylene blue is a water-soluble thiazine dye used to treat various conditions, which has been found to have unique analgesic property through temporary disruption of anal sensory nerve terminals of patients. Methylene blue has been used to treat intractable pruritus around the anus (9) and pain after hemorrhoid surgery (7). There is a latency period of 4–6 h for methylene blue to exert its analgesic effect after subcutaneous injection. Because methylene blue destroys the nerve myelin sheath during this period, the patient can feel burning pain. Therefore, in this study, we prepared methylene blue and ropivacaine in a certain proportion and used the nerve block effect of ropivacaine to cover the latency period of methylene blue so that the early burning pain of the patient after subcutaneous injection of methylene blue can be greatly reduced. Because methylene blue destroys the nerve myelin sheath, it has a long term analgesic effect, which may also cause anal sensation incontinence, perianal necrosis and other risks when used in high concentrations. However, the concentration of methylene blue for perianal injection is still controversial, and some researchers use concentrations are in the range of 0.2%–0.5%(7, 26–29), but there are also reports of higher concentration (30).

This study shows that the VAS pain score and total analgesics consumption within 14 days in group A and group B were significantly lower than those in group C, but the differences between group A and group B were not statistically significant, indicating that 0.1% and 0.2% methylene blue perianal injection have the same analgesic effect in the treatment of post-hemorrhoidectomy pain. There was no significant difference in complications among three groups, including urinary retention, secondary bleeding, perianal incision edema, and skin infection. At one month after the operation, the Wexner scores of group B were significantly higher than those of group A and group C, but the differences between group A and group C were not statistically significant, while the Wexner score among three groups decreased to zero at three months after operation. This indicates that 0.1% or 0.2% methylene blue subcutaneous injection after hemorrhoidectomy has little effect on anal function, and it is temporary and reversible. In addition, 0.1% methylene blue subcutaneous injection can not only effectively relieve pain, but also has less impact on anal function, with a lower risk of anal incontinence and higher safety. At the same time, subcutaneous injection of methylene blue around the perianal incision after surgery also conforms to the concept of preemptive analgesia.

However, there are still several limitations to be considered in the current study. First of all, this was a retrospective study with a small sample size and a short follow-up period of only 3 months. We should continue to expand the sample size and conduct long-term follow-up analysis of patients. More data are required to reduce the difference. In addition, more large-scale prospective randomized controlled trials should be carried out in the future to provide higher level of evidence.

## Conclusions

This study demonstrates that perianal subcutaneous injection of 0.1% and 0.2% methylene blue has comparable analgesic efficacy in treating post-hemorrhoidectomy pain, but 0.1% methylene blue is safer.

## Data Availability

The datasets presented in this study can be found in online repositories. The names of the repository/repositories and accession number(s) can be found in the article/supplementary material.
